# Unlocking maintenance—architecting STEP for maintenance and realizing remountable magnet joints

**DOI:** 10.1098/rsta.2023.0415

**Published:** 2024-10-09

**Authors:** Adrian van Arkel, Chris Lamb, Harry Robinson, Yannik Dieudonné

**Affiliations:** ^1^ UKAEA (United Kingdom Atomic Energy Authority), Culham Campus, Abingdon, Oxfordshire OX14 3DB, UK

**Keywords:** STEP, remote maintenance, remountable magnet joints

## Abstract

The architecture of the Spherical Tokamak for Energy Production (STEP) has been developed to enable a hybrid maintenance approach using ports in the vacuum vessel for a limited list of tasks that must be performed shortly after shutdown, and larger openings to simplify and speed up major refits. Robotic handling systems in zero-human entry facilities will prevent workers from being exposed to the most hazardous environments. While the approach is largely grounded in existing technologies, the scale and environment of STEP will require significant technology development. Notably, programmes have been established to develop service connections and in-vessel robotic technologies. The engineering integration of the maintenance strategy into the tokamak remains a priority, as does ongoing work to simplify and reduce the cost of the buildings required to facilitate maintenance. Remountable magnet joints are critical to ensuring life-limited magnet components can be replaced during the STEP lifetime and realizing the STEP maintenance strategy. It is a high-risk endeavour owing to the low technology maturity of the potential solutions and owing to the tough and intertwined technical challenges and constraints imposed by both the fundamental physics and the STEP requirements and architecture. An integrated design approach has been taken to balance many competing factors and integrate with interfacing systems, and a multi-faceted technology development programme has been established to address technical risk and to inform, verify and validate the STEP remountable magnet design.

This article is part of the theme issue ‘Delivering Fusion Energy – The Spherical Tokamak for Energy Production (STEP)’.

## 1. Part 1—Architecting STEP for maintenance

### (a) Introduction

#### (i) The maintenance requirement

Efficient maintenance is a fundamental programme objective for the Spherical Tokamak for Energy Production (STEP) [1,2] necessary for maximizing the time available for powerplant demonstration purposes, increasing resilience to damage, enabling upgrades as technologies develop and paving the way to commercial operating regimes. The greatest challenge in achieving this objective lies in the tokamak, where the harsh environment will prevent human access, so all maintenance must be undertaken by specialist radiation-tolerant machinery. A viable maintenance regime requires the careful balance of many competing factors, so the work of the team is to identify a concept with opportunities and challenges that are tractable.

**Figure 1.**
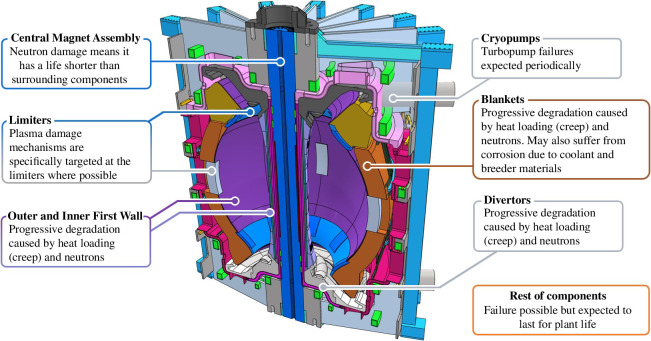
Main components of the STEP with expected failure modes.

Within the tokamak, there are five fundamental operations that fall under ‘maintenance’: inspection of the tokamak to ascertain its condition; recovery from unplanned failures; replacement of components at the end of their design life; upgrading components in line with the STEP operating phases; incorporate technological developments. Within the tokamak, there are many components that must be maintained, see figure 1.

#### (ii) The state of the art

Maintenance of tokamaks has been undertaken for decades, but the conditions in the STEP are very different to its predecessors. Existing tokamaks, including the recent Mega Amp Spherical Tokamak Upgrade (MAST-U) in the United Kingdom and the Japan Torus−60 Super Advanced, are designed for ‘hands-on’ maintenance. This places significant constraints on the type of fuel they can use, and therefore their power. Only two tokamaks have been operated with the STEP proposed power-plant fuel mixture of deuterium–tritium (DT), the Tokamak Fusion Test Reactor (TFTR) in the United States and the Joint European Torus (JET) in the United Kingdom. The DT fuel mixture produces high energy (14 MeV) neutrons that will make the structure of the tokamak radioactive. Both TFTR and JET had tight neutron production limits to prevent significant build-up of radioactivity, but the latter was also fitted with a robotic handling system in the late 1990s to minimize the need for humans to enter.

The ITER tokamak currently under construction in France is designed to operate with far higher neutron budgets that get closer to the STEP power-plant conditions. Once in DT operations, ITER will be maintained and upgraded entirely by robotic systems that have their roots in the JET systems, using horizontal ports.

Beyond fusion, there are many sources of inspiration for the STEP maintenance strategy and technology choices. For example, in the fission industry: components to be handled regularly, such as fuel pins, are standardized to simplify logistics; handling kinematics around the harshest reactor environments is intentionally kept simple; interim decay stores allow radiation levels to subside before complex operations are undertaken; shielding and remote processing equipment are used to protect operators and specialized technologies have been developed to work in high radiation fields. The STEP programme is seeking to deploy similar techniques but faces the added challenge that much of the tokamak core requires periodic replacement, resulting in the need to handle larger and more diverse components than often associated with fission.

Modern scientific spallation neutron sources, such as the European Spallation Source (ESS), can also provide relevant case studies. At ESS, a diverse range of modules weighing up to 20 tonnes will become highly radioactive and require periodic vertical replacement from the target monolith. Parts of the ESS waste processing facility will be permanently inaccessible, with windowless shielding walls protecting those outside and with state-of-the-art radiation-hardened robotic, processing and safety equipment inside [3].

The STEP maintenance strategy has also been influenced by the petrochemical industry, which has developed elegant techniques for precisely manoeuvring modules that are significantly larger than the modules proposed for STEP. Techniques for making and breaking service connections are also of direct relevance to STEP.

A common theme through fission, neutron sources and the petrochemical industry is that the kinematics of handling difficult components in challenging environments should be simplified as far as possible. To achieve this, STEP must first unlock access to the core of the reactor.

#### (iii) Unlocking access to STEP

The intertwined rings created by the poloidal and toroidal field (TF) coils of a tokamak create significant access challenges for maintenance [4]. This is compounded in spherical tokamaks such as STEP because the smaller circumference for a given power affords less room for access ports once the space claims of the coils and tokamak services are accounted for. However, reduced size also creates opportunity; a smaller reactor can be broken down into fewer blocks with fewer interconnections, thereby enabling reduced complexity of handling, aligning and connecting components within the harsh environment of the tokamak. Realizing this opportunity is contingent on finding a way to move large modules through the magnetic coil cage.

Methods have been proposed to intricately manoeuvre large modules through complex paths between the coils [5] with difficult kinematics, or to move intact coils out of the way during maintenance with very challenging plant logistics. Similar options for STEP were evaluated but discounted.

**Figure 2.**
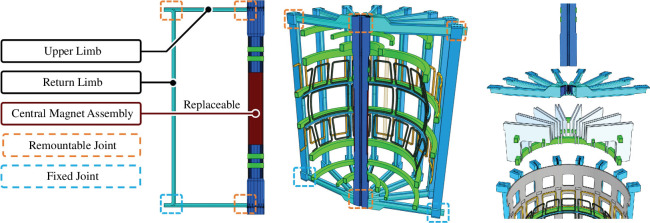
TF coil with three remountable joints (orange) with a fixed fourth joint (blue).

Instead, remountable joints (figure 2) will be included in the STEP TF coils for two key reasons:

— The central conductor of the TF coil requires periodic replacement, as the STEP aspect ratio results in a slender central column [2] restricting space for neutron shielding, thereby limiting conductor life.
— Removing the upper part of the TF coils allows the tokamak lid to be fully opened, creating a large opening through which annular core modules can be lifted.

With 16 TF coils, each with approximately 40 turns connected in series, the STEP segmented coil architecture involves 48 remountable magnet joints and approximately 2000 individual terminal connections—a failure of any single one causing a failure of the whole TF coil set—so the choice carries significant risk and technology challenges. The design development journey and associated challenges of the STEP magnet systems are described in [4], and part 2 of this paper describes the technology landscape, challenges and design development journey towards realizing remountable magnet joints for STEP.

#### (iv) Balancing many competing factors

**Figure 3.**
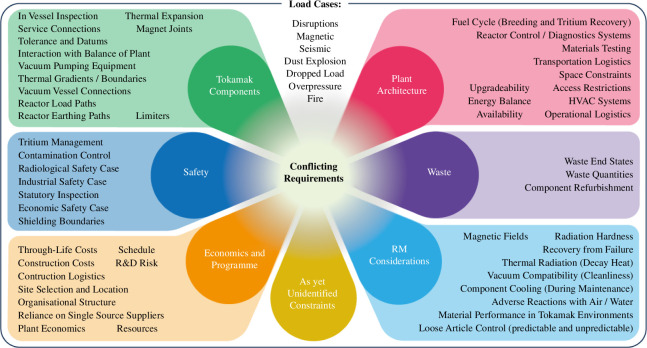
Competing factors in the maintenance of STEP.

There are many competing factors that must be balanced by a viable maintenance regime. A summary of factors that influence the maintenance strategy has been drawn up (see figure 3). With such a wide range of competing factors, it is not possible to satisfy all drivers and constraints to the maintenance challenge, and some are more influential than others. The following sections of this report highlight some of these tensions and the compromises required to resolve them.

#### (v) Hazard management during maintenance

During the tokamak assembly and early operation (i.e. before significant neutron generation), hazards are expected to be similar to those in other larger industrial plants, so proven techniques will be used to mitigate risks associated with confined spaces, high voltages, crushing, pressure and harmful materials. In later phases of operation (in small part after deuterium–deuterium operations, but primarily after deuterium-tritium operations) the tokamak structure will become radioactive [2,6].

The gamma dose rates two weeks after shutdown are expected to be in the range of 1–10 kGy h^−1^ inside the plasma chamber. The exact value will be highly dependent on the material choices for the plasma-facing and in-vessel components. Owing to radioactive decay this value will reduce with time, so it is possible to reduce the hazard and complexity of some operations by waiting several weeks, months or years.

Nevertheless, it will be necessary to handle and store radioactive material at STEP for many years following operation [7]. Workers and the public will be protected by purpose-built facilities that confine contamination, confine gases (in particular tritium) and shield radiation. Robotic handling systems will be used to ensure humans do not need to enter the most hazardous areas. An initial safety assessment has studied hypothetical worst-case failure modes, including dropped-load accidents and the collapse of buildings; these indicate that the resulting doses to workers and the public would be within stringent safety limits. Further detailed analysis will be completed as the programme develops.

### (b)The STEP maintenance scheme

#### (i) A hybrid approach

There are two principal ways in which STEP can be accessed, first by using the large opening created by the remountable magnet joints (referred to as vertical maintenance) and second by accessing through smaller ports between the magnetic coils (referred to as port-based maintenance). The requirements for STEP are broad enough that neither approach is sufficient alone, and therefore a hybrid maintenance approach is proposed.

Port-based maintenance will be used to undertake inspection and specific repair tasks (§1*b*(*iii*)). The principal reason for undertaking these activities through the ports is speed, accessing through the ports is possible without the time-consuming process of de-energizing (approximately two weeks), warming (approximately one month) and opening the coils (approximately three months).

The environment within the tokamak, however, will greatly restrict the capability of port-based systems, for three key reasons. First, the combination of ionizing radiation, residual magnetic field, thermal radiation and vacuum conditions within the tokamak will prevent the use of most electronics and complex mechanisms. Second, the ports through the STEP magnetic cage are highly congested by the services and supports needed for plasma operation, leaving very limited room for the deployment of maintenance equipment. Third, undertaking an extensive refit through the ports would be extremely slow, as large components will need to be subdivided significantly to fit through the ports and to remain within the payload of slender handling machines. The number of in-vessel service connections required would also have a significant negative effect on reliability—for this reason, as many connections as possible are to be made outside the cryostat.

Vertical maintenance is significantly less difficult from the standpoint of environmental and spatial challenges, thereby significantly reducing the complexity of a major refit, as well as minimizing the number of field coolant connections required. However, opening and closing the tokamak for vertical maintenance will take the better part of a year, so it is not practical for activities that must be completed quickly after shutting down the plasma.

There are a number of key drawbacks to the hybrid maintenance approach that the programme has consciously accepted. First, both approaches are hungry for space around the tokamak, resulting in increased building sizes and congestion challenges. Second, the tokamak must be designed to accommodate both approaches, adding further complexity. Third, the R&D challenges for port-based and vertical maintenance are quite different, so two distinct programmes must be established at additional expense. To minimize the effect of these drawbacks, the range of tasks to be undertaken through the ports will be restricted. Vertical maintenance is therefore considered to be the primary maintenance route for most of the tokamak.

#### (ii) Designing the tokamak for vertical maintenance

The remountable joints within the STEP TF coils will unlock access for maintenance by enabling the lid of the tokamak to be removed. This presents the opportunity to move the complexity of many maintenance tasks to less constrained areas and enables flexibility in how STEP can be upgraded. Realizing these opportunities requires careful architecting of the tokamak, and the maintenance strategy is therefore a fundamental pillar of the STEP design.

**Figure 4.**
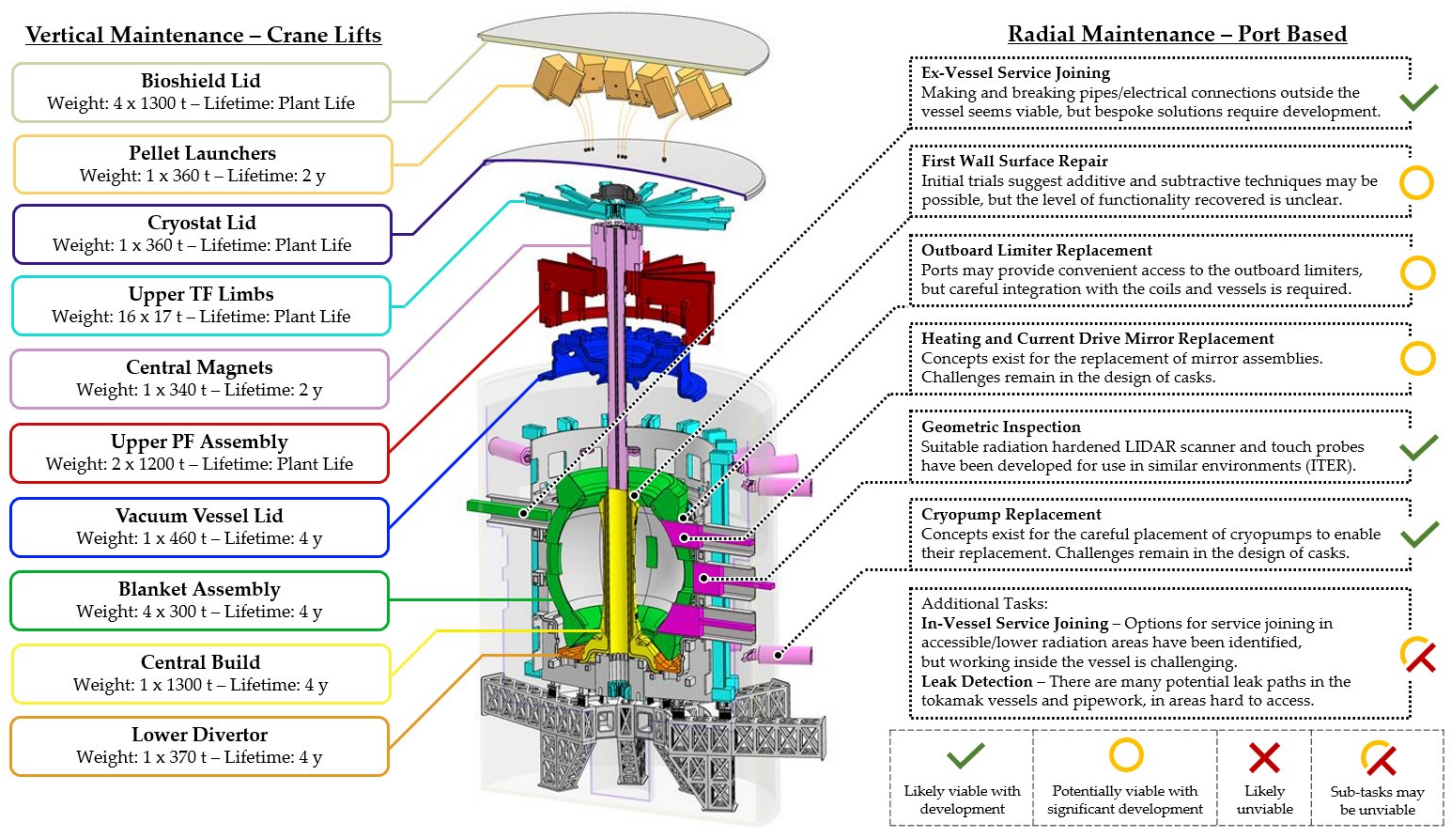
Tokamak vertical maintenance segmentation (left) and port-based capability proposals (right).

The way the tokamak is segmented is a complex compromise of factors including the viability of load paths, coolant routing, alignment tolerances, electrical conductivity and mechanical handling. Several segmentation approaches have been studied in detail by cross-disciplinary working groups, with the designs converging on the arrangement shown in figure 4. This arrangement requires a lifting capacity of approximately 1300 tonnes, plus an allowance for lifting adaptors.

The arrangement of the modules in STEP allows the service connections to be made from outside the tokamak, improving access and avoiding the challenges of making connections in the harshest environments. It is anticipated that this will enable resilient connection and disconnection of modules; an R&D programme has been established to confirm these benefits (refer to §1*c*(*ii*)). Attention is shifting to calculating the structural and thermal load paths of the modules, identifying methods of restraining modules, optioneering vessel seal arrangements and leakage budgets and the standardization of handling interfaces.

Once disconnected from their services and supports, modules will be lifted from the tokamak using a crane located in a hot cell from above and then transported to a logistics facility adjacent to the tokamak building for interim storage and processing (refer to §1*b*(*iv*)).

#### (iii) Designing the tokamak for port-based maintenance

There is a clear benefit in being able to inspect and repair the tokamak soon after shutdown, which necessitates working in extremely harsh and congested environments to avoid the time taken to return the tokamak to less onerous conditions.

Even before DT operations activate tokamak components, a challenging mix of requirements will exist for port-based maintenance equipment. There be a requirement to: manage the heat radiated from tokamak components that are held at 600°C; operate in an inert atmosphere to avoid oxidation damage to hot components; operate in moderate residual magnetic fields in the milli Tesla range and potentially high fields of multiple Tesla if the activity is to be carried out before the TF coils are ramped down (an activity taking several weeks). In the latter high-powered DT phases of the operation of STEP this is compounded by the tokamak background dose rate (1–10 kGy h^−1^ in the plasma chamber, two weeks after shutdown), which will rule out the use of many electrical items owing to the adverse effect on semi-conductor chips and electrical insulation.

A detailed study of the tokamak has been undertaken to identify candidate port-based maintenance activities and assess their viability. Tasks identified as viable are incorporated into the design baseline, and those having significant showstoppers are explicitly excluded. Several tasks are identified as potentially viable and shown in figure 4; ongoing studies are assessing the benefits and drawbacks of including those tasks in the design baseline.

In all phases, the port sizes will significantly restrict access and therefore payloads. Limiter [8] replacement is a key capability (plasma damage mechanisms are specifically targeted at limiters where possible) and is expected to take several months. Having this capability means that dedicated large ports for maintenance alone have been ruled out to avoid exacerbating congestion of tokamak service routing and reducing space available for tritium breeding [9]. Smaller penetrations within the limiter for less capable deployments are also intended.

A recurring issue is that of coolant routing within the tokamak. All components in the tokamak have integral cooling pipework that would need to be connected and disconnected during replacement. Undertaking these activities in the heart of the tokamak would provide great flexibility in the replacement of damaged components but it has been provisionally ruled out for reasons including the challenge of isolating and partially draining parts of a coolant system; neutron damage making any welded or mechanical seal inherently unreliable; tooling to make and break the joint is generally complex and/or intrusive; testing to verify joint quality is extremely challenging and rectifying poor quality joints is essentially impossible. The intent is, as far as possible, to connect coolant lines to the components from the outside only, as shown in figure 4.

#### (iv) Facilities to enable maintenance for STEP

**Figure 5.**
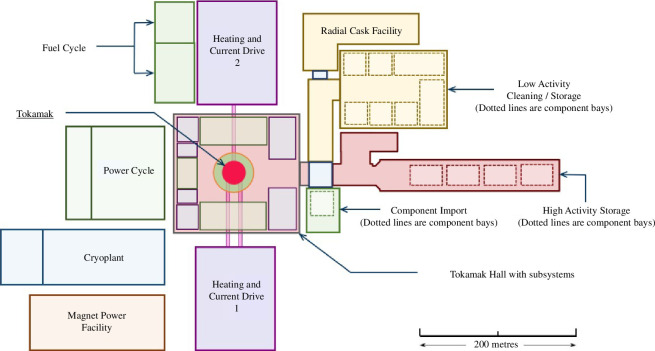
Tokamak complex including the tokamak hall and TLC with the tokamak highlighted in red.

The hybrid maintenance scheme requires significant space, loadbearing capacity and hazard management in the region around the tokamak. The buildings and facilities around the tokamak are therefore considered an integral part of the tokamak maintenance concept. Feasibility studies have concluded that two key facilities are required: the tokamak building will include hot cells to enable access to the tokamak and a Tokamak Logistics Centre (TLC) will be used to prepare new tokamak modules and process used modules (see figure 5).

For port-based maintenance tasks (figure 4), initial studies have found 40 ft ISO container-sized casks to provide sufficient space to house tooling and tokamak modules while also being viable from logisticical and spatial perspectives. Challenges remain with tokamak docking processes and the logistics of performing tasks under vacuum.

For vertical maintenance, two key decisions have been taken. First, the segmentation of the tokamak outlined in §1*b*(*ii*) has been driven to reduce the size and payload requirement of buildings as far as possible, but modules are still cumbersome. Second, the positioning of a hot cell above the tokamak will provide confinement and shielding functions, as the practicalities of using casks to transport and lift a wide variety of large components in the vicinity of the tokamak are prohibitive. These decisions have significant implications from a building perspective, so feasibility studies have been undertaken in the following areas: outline load path calculations; shielding thicknesses; process flow and HVAC/contamination control systems. These studies have directly influenced the segmentation outline in §1*b*(*ii*), and initiated further studies to characterize the rate of off-gassing and dust production from spent tokamak components to inform future decisions around atmospheric control and encapsulation of components.

The size of the TLC is driven by the tokamak size and the complexity of operations to be undertaken. Initial studies of the site layout concluded that the TLC can only occupy one side of the tokamak building because the remaining perimeter is required for power cycle, fuel cycle and plasma support systems. This complicates the TLC layout, as one building footprint must accommodate a variety of use cases in different phases of the programme [2], from initial assembly through to decommissioning.

A major refit of the tokamak core involves extensive decommissioning of spent components and assembly of fresh components. Therefore, many of the processes required for maintenance are analogous to initial assembly and end-of-life decommissioning, meaning the same equipment and infrastructure can be used. It is recognized that initial construction and the latter phases of decommissioning will require human access to areas that are out of bounds during normal operation, and this requirement is factored into initial building concepts.

The scale and complexity of the buildings to support maintenance remain a significant concern. Priority tasks are ongoing to develop a more complete understanding of fundamental drivers, ease requirements, refine building layouts and reduce the cost of their construction.

### (c) Maintenance enabling technologies

#### (i) Heavy load handling

Techniques for handling modules similar in size and weight to those in the STEP design are well developed but must be adapted for use in the harsh and congested STEP environment. A priority for the team is to ensure that the kinematics and lifting interfaces of the tokamak modules are kept simple, thereby ensuring the principal challenges are in engineering design rather than technology R&D. Nevertheless, radiation-hardened sensors, actuators and infrastructure will be necessary to operate in the harsh latter phases of operation.

#### (ii) Service connections

Developing a robust, repeatable and efficient means of making and breaking service connections is essential for viable tokamak maintenance. The architecture of STEP is being developed to place joints in more accessible and less harsh regions but factors including congestion, radiation rates, vacuum compatibility and inability for human access compromises positioning and necessitates new techniques. The challenges are amplified by the diversity of connection types in STEP, which include high- temperature and pressure gases, high- temperature and pressure liquids, cryogenic liquids, electrical, optical and vacuum joints.

With coolant selections and space allocations becoming firmer, it is now possible to embark on the preliminary designs of service connections. Space has been allocated in the cryostat and vacuum vessel ports to enable services to be passed to the in-vessel components from the tokamak perimeter, with bespoke machinery being necessary to form nested vacuum, pressure, optical and electrical connections. The residual dose rate in the ports is several orders of magnitude less than inside the tokamak, therefore creating the opportunity to use more dexterous machinery and cameras.

The strategy is to use gasket seals rather than welded connections, thereby reducing the risk associated with welding thick-walled piping, limited space availability for tooling, welding neutron-damaged material and qualifying welds/rectifying failed welds. A testing programme has been established to characterize the performance of gasket seals in STEP relevant conditions, to form the foundation of upcoming concept work. The results from this will be used to inform requirements for tolerable leakage rates, pumping capacities and engineering designs.

#### (iii) In-vessel robotics

The task list for in-vessel robotics is intentionally restricted (see figure 4), thereby reducing the R&D challenges associated with developing systems to operate in the highly onerous STEP environment. There remains a significant engineering challenge to integrate the planned tasks into systems suitable for the congested and harsh environment of the STEP. The scope of this work would grow significantly if one or more of the ‘potentially viable’ tasks become necessary, so finalizing the list of tasks in order to fully scope the R&D programme is a priority.

## 2. Part 2—Realizing remountable magnet joints

### (a) Remountable magnet joint fundamentals

As described in §1*a*(*iii*), the principal function of the remountable magnet joints is to enable the replacement of the in-bore magnet components (inner TF limbs, central solenoid and S-coils), which are expected to receive a critical radiation dose well before the end-of lifetime of the plant and to enable a vertical maintenance regime that will vastly improve plant availability over port-only-based maintenance systems. By splitting and rejoining (remounting) segments of a superconducting coil, greater access can be granted to the machine for bulk component handling, and segments of the coils that are subject to irradiation damage that can be replaced.

Superconducting magnets are typically wound in continuous coils, benefitting from zero-resistance current flow that can be used to generate high fields, but they must be kept at cryogenic temperatures and within stringent mechanical limits to retain these zero-resistance, high-field superconducting properties [4]. Breaking the coils has several effects that need to be mitigated, including: field discontinuity; current redistribution between superconducting elements (or tapes) in a cable; and heat generation from resistive current flow, which can give rise to a thermal discontinuity.

The defining feature of a remountable magnet joint is a breakable interface that can be repeatably disassembled and reassembled over the lifetime of the device. This is an extension of the demountable joint concept, which only implies the ability to disassemble a coil, for example, to replace a damaged coil. Remountable joints can be broadly classified according to three high-level features: the type of conductor connected via the joint (superconductor or resistive material); the mechanism through which the remountable connection is made (mechanical contact or soldering); the level of the conductor architecture at which the remountable connection is made (at the wire, whole cable or cable array level).

**Figure 6.**
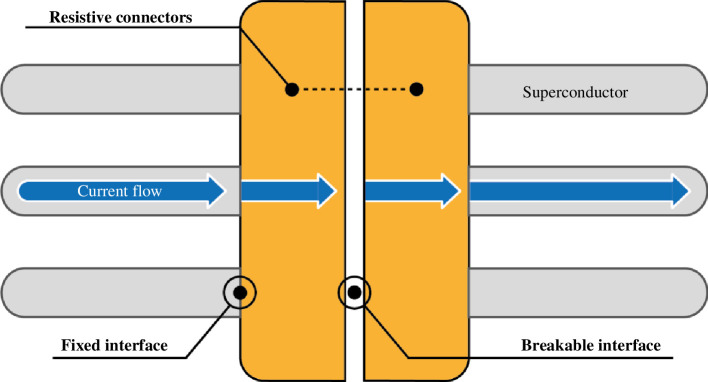
Diagram showing current flow across a remountable magnet joint.

A high-level diagram of a potential remountable joint arrangement is shown in figure 6, where current flows from a superconducting cable, into a resistive terminal, across a breakable interface, into the opposing terminal and then finally back into a superconducting cable. Each step contributes electrical resistance, which generates heat that needs to be removed. The importance of ensuring low resistance is further explained in §2*c*(*i*).

### (b) Remountable magnet joint technology landscape

Fixed joints that are made once and last the lifetime of a device have been developed for a range of superconducting magnet applications, including magnetic resonance imaging scanners [10]. These typically facilitate the assembly of devices and join feeder cables to coils and are challenging in their own right, but remountable superconducting magnets are far less mature.

Pressed and soldered contacts for cryogenic applications are a relevant area of ongoing research, as they play a crucial role in managing heat loads and hence cost and performance of cryogenic equipment. Pure copper pressed contacts are suboptimal for remountable joints, as they have a propensity to tarnish, making them very sensitive to environmental and processing conditions leading to large variability in contact resistance [11]. This would make it very difficult to establish reliable, repeatable connections without resorting to very high applied pressures of hundreds of MPa [11,12]. Materials that show promise for cryogenic contacts and have a good application track record are silver, gold, indium or a combination thereof [11–14].

At present, there are tokamaks that make use of remountable joints but it should be noted that all of them employ resistive magnets. Alto Campo Toro (Alcator) C-Mod and MAST-U make use of feltmetal sliding joints [15,16], which as the name suggests, allow the mating faces to slide relative to each other. This has advantages in terms of load handling, assembly and geometric tolerances but comes at the cost of increased joint resistance. Both NSTX and ST40 both employ static joints with compliance features in the radial limbs to mechanically decouple the radial limbs from the centre column [17,18], while DIII-D with its static joints has no such compliance features [19]. Notably, Compact Assembly Upgrade, currently under construction at the Czech Academy of Sciences, uses both static joints at the midplane and feltmetal sliding joints between the upper radial limbs and centre column [20]. While resistive remountable joints for tokamaks appear to be relatively mature, most of the aforementioned machines have undergone design iterations of their joints after commissioning either because of failures in operation or upgrades to improve performance [16,21,22]. Resistive remountable joints, while significantly different from superconducting ones, offer valuable opportunities for learning about the behaviour of the joint interface and implementation of the technology in a fusion environment, as well as operational and manufacturing experience.

To the STEP team’s knowledge, the first proposal for demountable superconducting magnets for tokamaks is the demountable externally anchored low stress magnet system [23]. Since this initial proposal, there have been a large number of design studies and proposals with the first mention of a remountable high-temperature superconductor (HTS) fusion reactor in 2002 [24]. Despite this, there are currently no operational tokamaks with remountable superconducting magnets, but there are examples of joint prototypes and magnet systems outside of tokamaks. The requirements for low-temperature superconductors (LTS) and HTS are very different but previous work on LTS remountable joints is nonetheless relevant for design inspiration, as both variants have a need for a low-resistance connection between joint terminals.

The HTS remountable joints, owing to their greater stability compared with LTS, permit the operation of resistive joints at intermediate temperatures around 20 K and fields of several Tesla [25]. The termination of the HTS cables, particularly those made from rare-earth barium copper oxide (REBCO)-coated conductors, is different from LTS and requires the development of different manufacturing techniques and joint geometries. One key area of research is making low-resistance solder connections with REBCO tapes. This is challenging, as the properties of REBCO-coated conductors are known to degrade under prolonged exposure to elevated temperatures during solder impregnation [26,27] and joint resistivities are dependent on the batch of superconductor used, even from the same manufacturer [28]. The state of the art in soldered lap joints between single superconducting tapes achieves joint resistivities of the order of tens of nΩ cm^2^ [29], but it remains difficult to scale these results to larger structures.

Nonetheless, there are multiple physical examples of cable-scale soldered REBCO-coated conductor terminations and demountable joint prototypes in the literature. Most feature a REBCO cable soldered into a copper terminal, which is then connected to a second terminal through a remountable interface. There is the notable exception of joint development for stacked tapes assembled in rigid structure conductors for a future heliotron device where the joint is made between individual tapes in a full-sized cable [30] among other less well-developed concepts. The most advanced magnet prototype demonstrating the use of demountable joints is probably the recent TF model coil tested by Commonwealth Fusion Systems [31].

### (c) Major challenges

**Figure 7.**
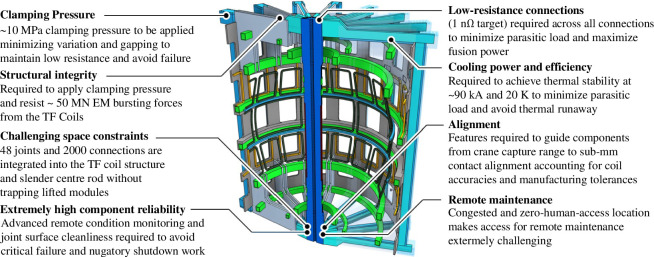
Remountable magnet joint major challenges.

The requirement to repeatably break and remake superconducting magnet connections over the lifetime of a device brings a whole host of challenges: some can be resolved by good design; most will require a significant R&D programme to fully overcome them. And some are imposed by the STEP design choices that trade various factors to optimize the design for the requirements of STEP. The congested and irradiated environment of a tokamak requires the integration of remountable magnet components among a whole host of other equipment [2] and all operations have to be performed remotely. The principal challenges associated with the STEP remountable magnet joints for the TF coils are shown in figure 7.

#### (i) Low resistance

STEP is aiming to demonstrate fusion power plant viability by exporting net power to the grid, making it extremely sensitive to parasitic loads [2]. The primary challenge with realizing remountable magnets for a fusion power plant is to minimize electrical resistance, as this has a direct effect on the cooling power required to combat ohmic heating. Magnet joint resistance is a function of: the resistivity of the materials, which varies with temperature; the current path through these materials; and the effective area of current transfer.

Given that ohmic heating is proportional to the square of the current, the power dissipated in the STEP remountable joints at the high operating current of approximately 90 kA may be significant. The operating temperature for the STEP magnets is 20 K, so when factoring in thermodynamic and cryogenic system efficiencies and losses, the power consumption of the cryoplant will be many times greater than the ohmic heating power, thereby adding to the parasitic load and eroding net fusion power [32]. This relationship is illustrated in table 1.

**Table 1.** Remountable joints ohmic heating and cryogenic cooling power requirement.

connection resistance (nΩ)	1	2	5	10	30
approximate total ohmic heating (kW)	16	31	78	155	466
approximate cryoplant power requirement (MW)	1	2	5	11	33

Minimizing resistance is also of paramount importance to the stability of the magnet coils, where there is a critical risk of quenching if local heating from the joints is not controlled [4]. In light of these driving factors, a target of 1 nΩ per connection has been set in advance of a fully defined operating window, while the cooling system has been designed to accommodate up to 5 nΩ of ohmic heating, at which point the cryoplant power requirement becomes significant.

#### (ii) Space

**Figure 8.**
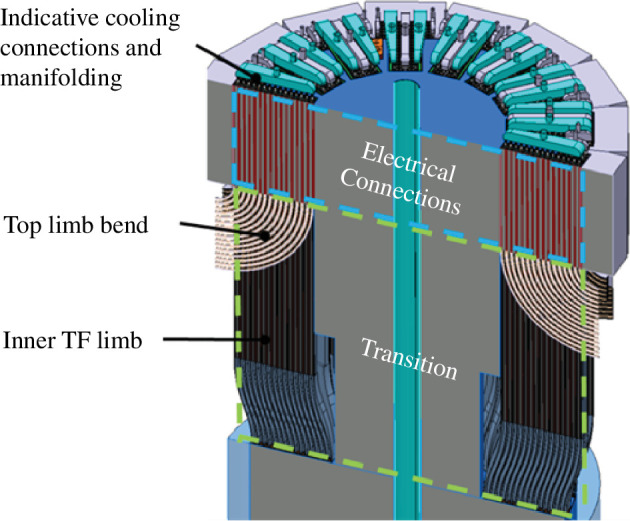
STEP centre rod with compact inboard magnets and remountable joints that must fit within the bore of the tokamak to enable vertical maintenance.

These challenges are made harder still by the STEP architecture and constraints, in particular the spatial constraints imposed by the narrow inboard build of STEP arising from the aspect ratio of the spherical tokamak and the vertical maintenance approach, which means lifted modules cannot protrude beyond their lifting aperture (figure 8).

#### (iii) Reliability and repeatability

The joints must also be extremely reliable for good plant availability. The 48 magnet joints are comprised of approximately 2000 individual connections; failure or excessive heating in any single connection will prevent field being generated by the TF coils, thus halting plasma operations. Furthermore, an intervention to repair a magnet joint could take months owing to the maintenance challenges described in part 1; worse still, it risks damaging the plant if a high-energy magnet quench ensues. The challenge of quench management and detection for the STEP magnets is discussed in depth in [4].

### (d) STEP remountable magnet design

#### (i) Design methodology and decision making

**Figure 9.**
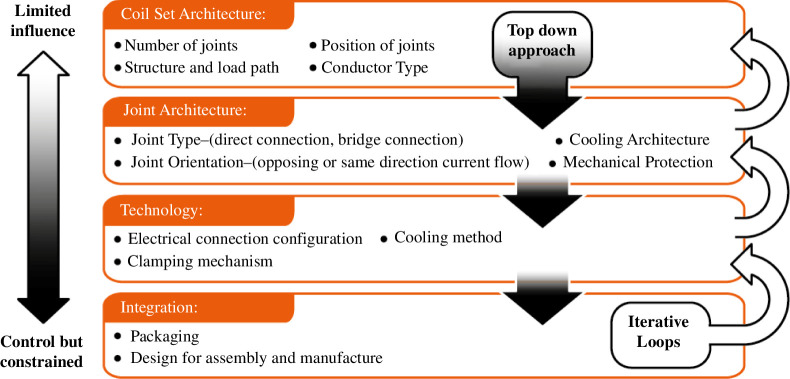
Remountable magnet joint decision hierarchy map.

The remountable magnet joints form part of the TF coils [4] and must be integrated into the wider STEP machine. The design space for these joints is therefore heavily coupled with the wider TF requirements and overall machine design where there are many requirements in tension. The design team has a varying level of influence over key design choices, depending on how they affect the plant design and STEP's measures of effectiveness [2]. Design decisions have been made by working from the top down with iterative loops for self-consistency and plant integration with the intent of finding the best compromises—not always the ‘best’ solution for a specific problem. Figure 9 shows the design and decision space for the remountable magnet joints going from plant-level architecture decisions with a wide stakeholder set to detailed design aspects that can be made in a tighter group.

**Figure 10.**
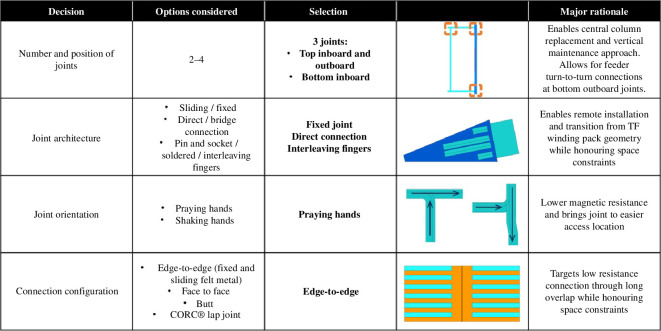
Architectural options, selections and principal rationales.

By working through this decision space using option identification, evaluation and down-selection techniques as appropriate, a design concept, summarized in figure 10, starts to form.

A praying-hands joint orientation has been chosen to minimize the magnetic field experienced by the joints caused by current flow in the same direction. This architecture also positions the joints in an accessible location for remote assembly and maintenance.

The remountable interfaces use pressed contacts rather than alternatives such as soldered connections owing to concerns over the ability to repeatably break and remake soldered joints by remote means without damaging the joints. Bulk connections are made in each joint via interleaving fingers whereby all of the approximately 40 connections are made simultaneously, providing a spatially efficient method of overlapping the mating connectors for low resistance.

‘Edge-to-edge’ connections refers to the orientation of the REBCO tapes, with tapes positioned edge-on to the remountable interface. This allows current to transfer from tapes along the full electrical joint length with a minimal resistive path length and therefore resistance.

#### (ii) Design development

The design has been matured beyond this decision set by integrating these choices around the TF coilset [4] and interfacing plant, eliciting requirements and constraints on each component and developing the design with iterative analysis against this requirement set. Some notable features arise from this integration effort and are described in the following paragraphs.

The upper and lower centre rod joints are flared to a larger radius at the joint via the transition region seen in figure 8. This increases space available in the joint region to accommodate the required joint functionality, including a clamping device, overlapping connectors, alignment features to facilitate remote assembly and cooling inlet/outlets.

To achieve precision alignment and prevent gapping or jamming owing to manufacturing and assembly tolerances, a compliance region has been incorporated, as seen in figure 8. This region enables a small amount of carefully controlled flex in the joint conductors and radial plates leading to the electrical connection region, allowing them to conform to the desired aligned position under clamping loads without exceeding the stress and strain limits of the REBCO superconducting tape [4].

**Figure 11.**
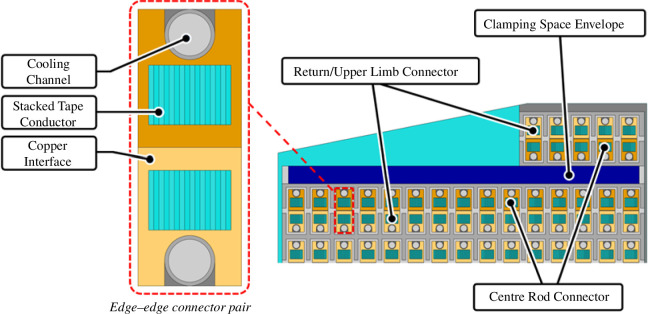
Plan view of remountable joint electrical connection region, with a detailed view of edge-to-edge connector pair.

The detail of the edge-to-edge connections, which comprises of approximately 230 stacked HTS tapes is shown in figure 11. The copper interface protects the HTS tapes from mechanical damage that would occur as the connectors are pressed together, allowing the connection to be re-made multiple times.

#### (iii) Ongoing design development

Some critical aspects of the design are still being defined:

— The pressed contact method requires careful selection of bulk material, finishes and coating of the connection interfaces for low contact resistance and good operational resilience. A copper-to-copper interface is specified as the reference but this *interface definition* will be informed by investigations as outlined in §2*f*.
— The *clamping mechanism* that applies pressure to the connection interfaces is critical for a low-resistance connection and requires that a pressure of 10–25 MPa be applied. There are many different ways that this pressure can be applied, such as hydraulics or thermal contraction of materials; but the space constraints, environmental conditions, reliability requirements and need for any actuation to be applied remotely make this particularly challenging. A number of designs have been developed aiming to satisfy these conditions, all of which have their own strengths and weaknesses; a down-selection process supplemented by proof of principle tests is planned to select the design that best balances a weighted requirement set.
— *Structural connections* must be made at each of the 48 remountable joints, each one with a different load case and set of spatial constraints. Large electromagnetic forces of the order of mega Newtons are induced in the TF magnets such that the structural connections must divert load around the electrical joints, keeping the stresses and strains within acceptable limits for the HTS conductors. A dedicated structural load path has been developed via the inter-coil structure and centre rod, the development and analysis of which are presented in [4]. A remotely made structural connection must be designed to honour this load bypass scheme.
— An *alignment scheme* has been developed that will guide replacement magnet limbs into a capture range into which vertical handling systems can deploy, gradually eliminating degrees of freedom and bringing joint faces into close sub-mm alignment. Alignment features are being developed and rapidly prototyped to realize this scheme.

### (e) Multiphysics modelling

**Figure 12.**
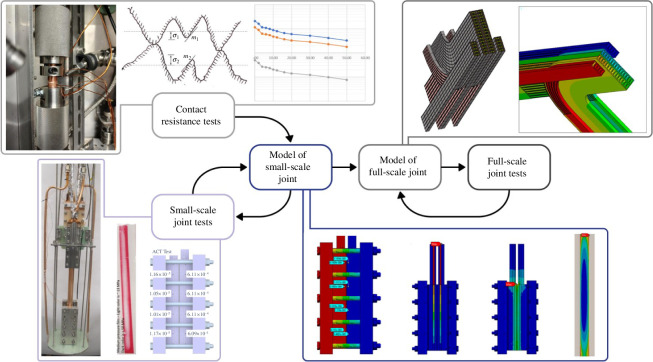
Workflow incorporating modelling with testing to reduce the burden of full-scale tests.

To reduce the burden of costly experiments, and to enable agile design, it is essential that simulations are performed to predict joint resistance. The fundamental physics at the contact interface between two metallic surfaces is complex—see top left of figure 12. The effective resistivity of a joint interface is much higher than that of the base metal because the conducting area across the interface is greatly reduced, as current can only flow between touching peaks.

Contact resistance is known to depend on several parameters, some of which are temperature dependent: contact pressure; Young’s modulus of the base materials; bulk resistivity; surface roughness; surface cleanliness; the presence of surface oxides. An expression for the thermal and electrical resistance of an interface can be developed from first principles [33], but predicting contact resistance requires knowledge of the properties of the surface oxides, which are typically unknown and highly variable, so some experimental testing is essential.

In commercially available simulation software, it is possible to perform coupled structural–thermoelectric analysis, with contact elements used at the interface to define a pressure-dependent resistance. If pressure-dependent resistance data are available from tests, these data can be used by the contact elements, and then the behaviour of a joint (where contact pressure varies along the joint) can be predicted. Once it can be shown that this modelling approach sufficiently predicts the behaviour of small-scale tests, then full-scale concepts can be analysed with confidence before committing to very expensive full-scale test programmes.

Figure 12 shows a workflow where small-scale tests and simulations are performed to build confidence in the modelling approach feeding into the design prior to the creation of full-scale models and validation tests.

The aim of this workflow is to identify the key parameters and relationships with iterative physical test and simulation cycles, ultimately leading to a fully predictive model that combines all key aspects of the joint behaviour. This model can then be confidently relied upon as the design develops, and used as the means to convey changes between experimentation, design, requirements and interfaces through a model-based systems engineering approach [34].

### (f) Technology development programme

Owing to the criticality, highly coupled dependencies, plant constraints, tough technical challenges and low technology maturity of some of the solutions described within this paper, realizing the STEP remountable magnet joints is a high-risk endeavour requiring a large and multi-faceted technology development programme. Some highlights of this programme include:

— *Connection prototyping* aims to demonstrate the functionality of the remountable magnet joints and the achievement of the key performance parameters. This iterative prototyping workstream is well underway in conjunction with advanced conductor technologies and has been invaluable in informing and verifying the design to date in conjunction with the multiphysics modelling described in §2*e*. As the STEP programme moves into the next phase of development, it will evolve and scale up to verify and validate the connection design whole joint designs and intended manufacturing methods.
— As the mode of pressure application is selected and its design develops, a *clamping development* workstream will commence with simple proof-of-principle tests to inform the design and understand mechanism behaviours at cryogenic temperatures progressing to prototype tests targeting and validating the approximate 10–25 MPa requirement.
— *Physics and operations testing* comprises a range of small, discrete tests to inform modelling and design and feeds directly into the multiphysics modelling described in §2*e*. Tests are targeted at aspects such as better understanding the electrical behaviour of the superconductor termination, exploration of factors that affect degradation such as multiple remounts, characterizing the pressure–contact resistance relationship described in §2*e* and plugging gaps in materials property data for the STEP magnet operating temperature of 20 K.
— To address the challenge of *remote alignment*, modular test rigs are being developed to enable testing of the staged alignment strategy and specific features to demonstrate remote alignment to sub-mm alignment and planarity. The modularity of the test rigs will enable variations in design to be accommodated arising from overall design evolution and feedback gained from the trials.

## 3. Closing remarks

The maintenance approach for STEP and the realization of remountable magnet joints are intrinsically linked. By taking on a hybrid maintenance approach with the vertical lifting of large modules for major refurbishments, port maintenance for faster repairs and inspection, and remountable magnet joints to extend the life of the magnets plus improve access, the STEP programme carries significant risk. While there are compromises, these choices will ultimately lift the ceiling on fusion plant availability and help in the quest for fusion power plant commercial viability. Collaboration will be essential in developing these technologies, and the authors are actively seeking opportunities to work with the wider community and industry.

## Data Availability

This article has no additional data.
